# Immune System Abnormalities in Schizophrenia: An Integrative View and Translational Perspectives

**DOI:** 10.3389/fpsyt.2022.880568

**Published:** 2022-04-25

**Authors:** Evgeny A. Ermakov, Mark M. Melamud, Valentina N. Buneva, Svetlana A. Ivanova

**Affiliations:** ^1^Laboratory of Repair Enzymes, Institute of Chemical Biology and Fundamental Medicine, Novosibirsk, Russia; ^2^Department of Natural Sciences, Novosibirsk State University, Novosibirsk, Russia; ^3^Laboratory of Molecular Genetics and Biochemistry, Mental Health Research Institute, Tomsk National Research Medical Center of the Russian Academy of Sciences, Tomsk, Russia

**Keywords:** schizophrenia, immune system, inflammation, cytokines, antibodies, T cell, B cell

## Abstract

The immune system is generally known to be the primary defense mechanism against pathogens. Any pathological conditions are reflected in anomalies in the immune system parameters. Increasing evidence suggests the involvement of immune dysregulation and neuroinflammation in the pathogenesis of schizophrenia. In this systematic review, we summarized the available evidence of abnormalities in the immune system in schizophrenia. We analyzed impairments in all immune system components and assessed the level of bias in the available evidence. It has been shown that schizophrenia is associated with abnormalities in all immune system components: from innate to adaptive immunity and from humoral to cellular immunity. Abnormalities in the immune organs have also been observed in schizophrenia. Evidence of increased C-reactive protein, dysregulation of cytokines and chemokines, elevated levels of neutrophils and autoantibodies, and microbiota dysregulation in schizophrenia have the lowest risk of bias. Peripheral immune abnormalities contribute to neuroinflammation, which is associated with cognitive and neuroanatomical alterations and contributes to the pathogenesis of schizophrenia. However, signs of severe inflammation are observed in only about 1/3 of patients with schizophrenia. Immunological parameters may help identify subgroups of individuals with signs of inflammation who well respond to anti-inflammatory therapy. Our integrative approach also identified gaps in knowledge about immune abnormalities in schizophrenia, and new horizons for the research are proposed.

## Introduction

All body systems are interconnected in varying degrees in health and pathology. The immune system is no exception. They are activated when interacting with an infectious agent or spontaneously affecting everything, including the central nervous system. In some cases, this can lead to pathological changes in human mental status. Schizophrenia, as an example of mental illness, is no exception. Ideas that inflammation and immunity are involved in the pathogenesis of schizophrenia have been around for decades, but they do not lose their relevance to this day (1). Back in 1930, Dameshek W. found quantitative changes in white blood cells in patients with schizophrenia (2). In 1937, Lehmann-Facius proposed the idea of a humoral autoimmune factor in the pathogenesis of schizophrenia (3). In 1976, E. Fuller Torrey and Michael R. Peterson proposed a viral hypothesis for schizophrenia, describing, among other things, the possible influence of concomitant immune perturbations (4). Over time, hypotheses about a direct immunocompetent culprit for the onset of schizophrenia have multiplied: general inflammation hypothesis (5), macrophage-T-cell hypothesis (6–8), autoantibody hypothesis (9), microglia hypothesis (10). In this systematic review, we summarized and systematized the available data on changes in various parts of the immune system in schizophrenia. The main question of this systematic review was the following: what changes and in what components of the immune system are found in schizophrenia and how are they associated with the disease?

## Brief Overview of the Immune System

**FIGURE 1:**
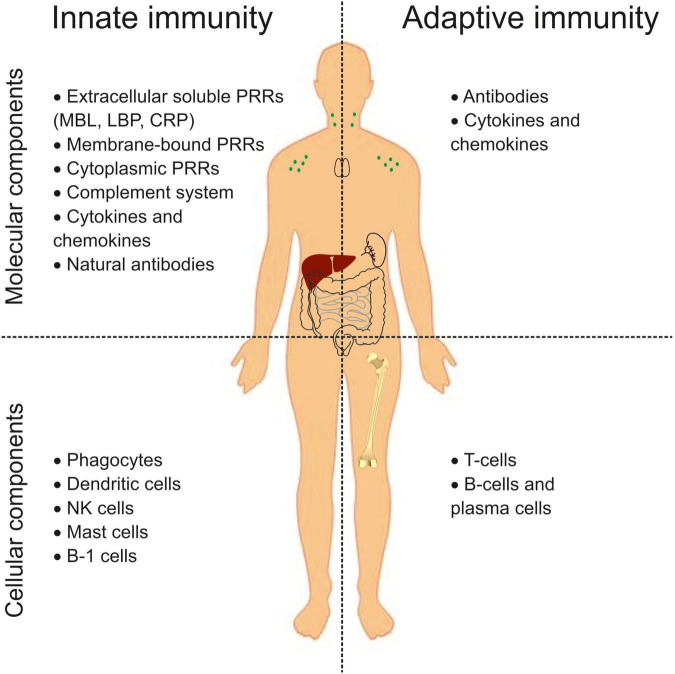
An overview of the innate and adaptive immune system components. PRRs, pattern recognition receptors; MBL, mannose binding lectin; LBP, lipopolysaccharide binding protein; CRP, C-reactive protein, NK, natural killer.

The immune system is generally known to be the primary defense mechanism against pathogens and has two arms: the innate (nonspecific) immune system and the adaptive (acquired) immune system (11). The mechanisms of innate immunity inactivate a significant part of pathogens. The adaptive immune response is activated when innate mechanisms are ineffective. Besides, innate immunity plays a vital role in response to tissue damage (aseptic inflammation), resulting in removing apoptotic and necrotic cells and tissue repair. The effector functions of these two arms provide molecular and cellular components (Figure 1). The nonspecific innate immune system recognizes pathogens through pattern recognition receptors (PRRs) (12). Extracellular soluble PRRs (mannose-binding lectin (MBL), lipopolysaccharide-binding protein (LBP), C-reactive protein (CRP), etc.) bind microbial products in liquid media and ensure their opsonization and phagocytosis. Membrane-bound and cytoplasmic PRRs recognize the pathogen-associated molecular patterns (PAMPs) or damage-associated molecular patterns (DAMPs) and initiate an immune response. Membrane-bound PRRs are mainly represented by Toll-like receptors (TLRs). Cytoplasmic PRRs include nucleotide oligomerization domain (NOD)-like receptors (NLRs), C-type lectin receptors (CLRs), retinoic acid-inducible gene-I (RIG-I)-like receptors (RLRs), etc. The complement system is involved in the opsonization of microorganisms and initiating inflammation. Natural antibodies bind exogenous and self-components and participate in their clearance. PAMPs or DAMPs sensing by PRRs engage cellular components in an immune response. The cellular components of innate immunity include phagocytes, dendritic cells, NK cells, mast cells, and B-1 cells. T-cells and B-cells are components of the adaptive immune system. B-cells, particularly plasma cells, produce antibodies, while T-cells provide recognition and destruction of cells carrying foreign antigens and regulate the immune response. Cytokines and chemokines orchestrate the effector functions of all components. However, this is a simplified representation. The division into cellular and molecular components is provisional, and many components are involved in both adaptive and innate reactions.

## Search Methods and Evidence Quality Assessment

**FIGURE 2:**
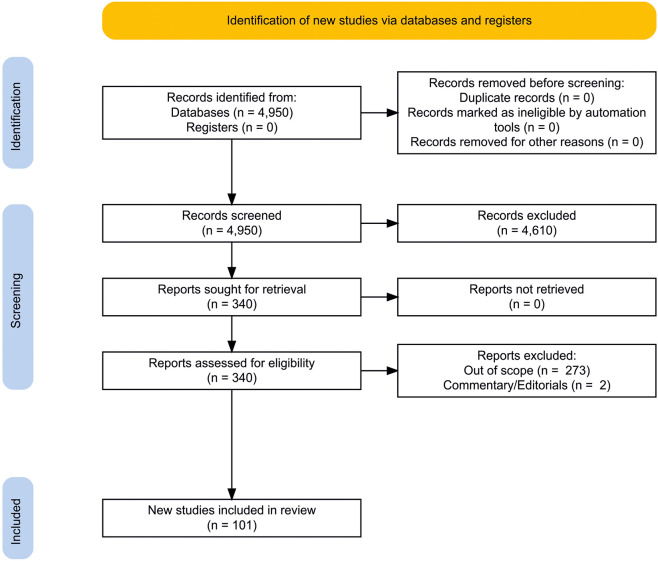
PRISMA 2020 flowchart diagram. Created using an online tool: https://estech.shinyapps.io/prisma_flowdiagram/ (15).

We followed the standards by Preferred Reporting Items for Systematic Reviews and Meta-Analyses (PRISMA 2020) (13, 14). We performed an electronic search using PubMed, Google Scholar, Scopus, and Web of Science to extract the necessary information from database inception to January 2022. The following basic search terms were used (both alone and in combinations): “schizophrenia,” “schizoaffective,” “psychosis,” ‘psychotic,” “meta-analysis,” “systematic,” “review.” The following additional terms were used in combination with the basic search terms: “immune system,” “inflammation,” “PRR,” “CRP,” “sCD14,” “LBP,” “MBL,” “TLR,” “NLR,” “complement,” “cytokine,” “chemokine,” “antibodies,” “antibody,” “IgG,” “IgA,” “IgM,” “natural antibodies,” “autoantibodies,” “*Toxoplasma gondii*,” “NMDAR,” “GAD65,” “gluten,” “cell,” “natural killer,” “NK,” “dendritic cell,” “neutrophil,” “eosinophil,” “basophil,” “monocyte,” “macrophage,” “microglia,” “lymphocyte,” “B cell,” “CD20,” “T cell,” “CD3,” “CD4,” “bone marrow,” “thymus,” “lymphatic,” “lymphatic nodes,” “spleen,” “liver,” “gut,” “intestine,” “axis,” “microbiota.” PRISMA 2020 flowchart diagram is presented in Figure 2 (15). One researcher reviewed titles and abstracts of the first 50 results of each query and then selected the reports. Automation tools to remove records were not used. Then two independent researchers (EAE and MMM) assessed the relevance of all articles retrieved. The final reference list was compiled based on the relevance of the topics covered in this review after the consensus of the researchers. Meta-analyses and systematic reviews were primarily included in the full text of the review. In their absence, case–control and/or cohort studies and/or case report were included. The main findings of this systematic review summarized in tables are based on 101 studies, including 33 meta-analyses, 5 systematic reviews and 63 other studies. However, the final bibliography contains more studies to broaden the context of the review.

The quality of the evidence found was assessed based on the risk of bias (the higher the risk of bias, the lower evidence quality). The following criteria were used to assess the risk of bias (Table 1), similarly, to Tanaka et al. (16) with some modifications. The risk of bias was assessed by two independent researchers (EAE and MMM). The final assessment of the risk of bias was approved after the consensus of the two researchers.

**TABLE 1:** Risk of bias evaluation criteria.

Risk of bias	Criteria
Low	Presence of more than two meta–analyzes without conflicting results
Moderate	Presence of at least one meta–analysis or systematic review with more than five case–control and/or cohort studies without conflicting results
High	No meta-analysis or systematic review, fewer than five case–control and/or cohort studies without conflicting results
Unclear	Presence of only case–control study, case reports and/or cohort study with conflicting results, meta–analyzes or systematic reviews with conflicting results; not enough data to assess the level of evidence

*According to Page et al. (14) with some modifications.*

## Alterations in the Innate Immune System in Schizophrenia

### Molecular Components of the Innate Immune System

Activation of the innate inflammatory response is mediated by a complex network of interactions between numerous molecular and cellular components of the immune system. Dysregulation of this complex network contributes to chronic inflammation. Available data on abnormalities in the molecular components of the innate immune system associated with schizophrenia are presented in Table 2.

**TABLE 2:** Abnormalities of the molecular components of the innate immune system in schizophrenia.

Changes	Risk of bias	References
**Pattern-recognition receptors**		
CRP levels are elevated in blood of patients with schizophrenia.	Low	(15–19)
Soluble CD14 levels but not LBP and MBL levels were elevated in schizophrenic patients compared with healthy controls.	Unclear	(20, 21)
Impaired TLRs expression in PBMCs has been identified in drug-naïve first-episode psychosis patients.	Unclear	(22–25)
Increased expression of NLRP3 and NLRC4 was found in the blood of patients with schizophrenia compared with healthy controls	Unclear	(26)
Complement system		
Serum levels of complement components C3 and C4 did not differ between schizophrenic patients and controls.	Moderate	(28)
**Cytokines and chemokines**		
Serum pro-inflammatory cytokines levels are elevated in schizophrenic patients compared to healthy controls.	Low	(31–36)
Cytokine levels differ in first-episode psychosis patients, acutely relapsed inpatients, and chronic patients. Cytokine levels are associated with the severity of clinical symptoms.	Low	(31–36)
Antipsychotic treatment significantly affects cytokine levels in schizophrenia.	Low	(37–39)
The levels of IL-1β, IL-6, and IL-8 are significantly elevated in the cerebrospinal fluid of patients compared to healthy controls.	Low	(40, 41)
Chemokine levels are altered in schizophrenia. IL-8 (CXCL8) is elevated in the cerebrospinal fluid of patients. MCP-1 (CCL2), MIP-1β (CCL4), eotaxin-1 (CCL11), and IL-8 (CXCL8) are elevated in serum of patients.	Low	(42–46)
**Natural antibodies**		
Antibodies to gram-negative bacteria, LPS, and *Saccharomyces cerevisiae* have been found in schizophrenia.	Moderate	(44)
Natural antibodies to cytokines/chemokines, tryptophan catabolites, and oxidative specific epitopes have been identified in schizophrenia.	Unclear	(45–47)
An increased level of natural antibodies with catalytic activity hydrolyzing DAMPs (nucleic acids, histones, MBP) was found in schizophrenia.	Unclear	(51–56)

*CRP, C-reactive protein; CD, cluster of differentiation; LBP, lipopolysaccharide binding protein; MBL, mannan-binding lectin; TLRs, toll-like receptors; PBMCs, peripheral blood mononuclear cells; NLRP3, NLR family pyrin domain containing 3; NLRC4, NLR family CARD domain-containing protein 4; IL, interleukin; LPS, lipopolysaccharide; DAMPs, damage-associated molecular patterns; MBP, myelin basic protein; MCP-1, monocyte chemoattractant protein 1; MIP-1β, macrophage inflammatory protein 1β.*

#### Pattern-Recognition Receptors

C-reactive protein is an acute-phase protein and one of the first discovered extracellular soluble PRRs. Several meta-analyses have confirmed that blood CRP levels are elevated in schizophrenia. In one of the first meta-analyses, Miller B. et al. showed that CRP levels were significantly elevated in patients with schizophrenia compared to healthy individuals (17). The prevalence of elevated CRP levels in patients with schizophrenia was 28%. Another study showed that CRP levels were moderately elevated in schizophrenia and that antipsychotic medication had no effect on CRP levels (18). Another meta-analysis also confirmed these observations (19). More recently, Osimo E.F. et al. reported that patients with high (>3 mg/L) CRP levels had a greater risk of psychosis compared to patients with low (≤3 mg/L) baseline CRP levels (pooled odds ratio was 1.5) (20). However, this association decreased after excluding participants with suspected infection (CRP > 10 mg/L). Besides, CRP levels have been associated with a risk of increased positive symptoms, cognitive impairment, microbiota disturbance, and metabolic syndrome in subjects with schizophrenia (21). Evidence for elevated CRP levels in schizophrenia has a low risk of bias (Table 2).

Other extracellular soluble PRRs have been less studied in schizophrenia. Soluble CD14 (sCD14) levels but not LBP levels were elevated in schizophrenic patients compared with healthy controls (22). The level of MBL did not differ between patients with schizophrenia and healthy controls (23). These data may indicate increased intestinal permeability in schizophrenia (see below).

Studies of membrane-bound PRRs are rare and controversial. There is evidence that TLR1, TLR2, TLR4, TLR6, and TLR9 expression levels in peripheral blood mononuclear cells (PBMCs) are reduced in schizophrenia, TLR3 and TLR7 levels are elevated, and TLR5 and TLR8 levels do not differ from controls (24). In another study, TLR5 and TLR8 expression levels in PBMCs decreased in drug-naïve first-episode psychosis individuals before and after treatment compared to controls (25). A lower functional activity of TLRs after agonist activation has also been shown. TLR3 but not TLR4 expression levels were higher in PBMCs of drug-naïve patients (26). The therapy did not affect the expression level of these receptors. In another study, increased expression of TLR4 and TLR5 in PBMCs was found in drug-naive patients (27). TLR4 expression returned to normal after treatment. Generally, further studies of changes in TLRs expression on various immune cells and their functional activity in schizophrenia are needed.

Studies of cytoplasmic PRRs are also rare in schizophrenia. A recent report showed that increased expression of NLRP3 and NLRC4, together with high baseline levels of circulating components of IL-18, was observed in the blood of patients with schizophrenia and bipolar disorder compared with healthy controls (28). Also, a correlation between the levels of cholesterol types in the blood and the expression of inflammasome system elements in these diseases has been shown. There is also evidence that NLRP3 and NLRC4 in microglia and astrocytes mediate sterile inflammasome activation and neuroinflammation (29). However, more research is needed. Data on the expression level of cytoplasmic PRRs and the activity of inflammasomes may expand our understanding of the activation of inflammatory pathways in schizophrenia.

#### Complement System

The complement system is involved in initiating the inflammatory response. A recent systematic review showed that the activity of the classical complement pathway did not differ between schizophrenia and controls (30). The meta-analysis results in this study showed that serum levels of complement components C3 and C4 did not differ between schizophrenic patients and controls. This evidence has a moderate risk of bias (Table 2). However, there is evidence of increased levels of C4 in the cerebrospinal fluid in patients compared with healthy individuals after adjusting for sex and age (31). Complement component C4 is known to mediate synaptic pruning (32). Therefore, the study of anomalies in C4 and other complement system components may be of translational importance.

#### Cytokines and Chemokines

Cytokines secreted by activated immune cells orchestrate the effector functions of the immune response. Although cytokines regulate not only innate but also adaptive responses, we have placed them in this section.

Changes in cytokine levels in schizophrenia are probably the most studied but controversial area of research in biological psychiatry. Numerous systematic reviews and meta-analyses have been published on serum cytokines in schizophrenia (33–39). Despite some controversy, all of these meta-analyses suggest cytokine dysregulation in schizophrenia and a predominance of pro-inflammatory cytokines in the serum of patients. Levels of interleukin (IL)-6, IL-1β, IL-12, tumor necrosis factor (TNF)-α, and transforming growth factor (TGF)-β were elevated in schizophrenia, while levels of IL-2, IL- 4, and IL-17 did not change (38). Cytokine levels differ in first-episode psychosis patients, acutely relapsed inpatients, and chronic patients. Also, cytokine levels are associated with the severity of clinical symptoms. Besides, antipsychotic treatment significantly affects cytokine levels (40–42). The levels of cytokines in the cerebrospinal fluid of patients are also altered. In particular, the levels of IL-1β, IL-6, and IL-8 are significantly elevated in the cerebrospinal fluid of patients compared to healthy controls (43, 44). Interestingly, IL-8 is a chemotactic cytokine or chemokine CXCL8 and promotes neutrophil and monocyte chemotaxis. IL-8 increased not only in the cerebrospinal fluid (43, 44), but also in the serum of first-episode psychosis patients and multiple-episode schizophrenia individuals, as confirmed by meta-analyses (45, 46). Among other chemokines, monocyte chemoattractant protein 1 (MCP-1 or CCL2), macrophage inflammatory protein-1β (MIP-1β or CCL4), and eotaxin-1 (CCL11) were also elevated in serum of patients (46). The serum levels of MIP-1α (CCL3), fractalkine (CXCL1) and interferon gamma-induced protein 10 (IP-10 or CXCL10) did not differ between patients and controls (46). Importantly, MCP-1 (CCL2) serum levels were higher in first-episode psychosis and multiple-episode schizophrenia, while MIP-1β (CCL4) and eotaxin-1 (CCL11) levels were higher only in multiple-episode schizophrenia (46). Thus, chemokine levels are also altered in schizophrenia (47).

Altogether, the available evidence for changes in the levels of cytokines and chemokines in schizophrenia has a low risk of bias (Table 2), so they can be considered reliable.

#### Natural Antibodies

Natural antibodies are germline-encoded immunoglobulins found in individuals without prior antigenic exposure (48). They are also characterized as oligospecific low-affinity immunoglobulins that recognize phylogenetically conserved epitopes. Natural antibodies bind exogenous (mainly bacterial) and self-components (apoptotic cell fragments, oxidative neo-epitopes, and other autoantigens, including DAMPs). Natural antibodies perform homeostatic functions and mitigate autoimmune and inflammatory responses.

Natural antibodies to intestinal bacteria have been found in patients with schizophrenia. In particular, according to a meta-analysis, IgA, IgM, IgG to gram-negative bacteria, LPS, and anti-Saccharomyces cerevisiae antibodies are present in patients with schizophrenia (49). This evidence has a moderate risk of bias (Table 2). The formation of such antibodies may be associated with gut dysbiosis and increased intestinal permeability. Data from this meta-analysis of increased blood levels of alpha-1-antitrypsin, lipopolysaccharide-binding protein (LBP), soluble CD14 (sCD14) also suggest an increase in intestinal permeability in schizophrenia.

Natural IgM and IgA to cytokines/chemokines, tryptophan catabolites (TRYCATs), NO-cysteinyl, malondialdehyde, and other oxidative specific epitopes were found in patients with schizophrenia (50). Changes in the level of these specific natural antibodies are associated with some clinical phenotypes of schizophrenia. For instance, patients with deficit schizophrenia exhibit increased IgA responses directed to quinolinic acid, picolinic acid, and xanthurenic acid and relatively lowered IgA levels to anthranilic acid and kynurenic acid when compared to patients with nondeficit schizophrenia (51). Increased production of IgA to TRYCATs was significantly associated with the severity of anxiety and depression symptoms in schizophrenia (52). Besides, deficit schizophrenia is accompanied by a significant decrease in IgM to TRYCATs (53). A decrease in natural IgM to malondialdehyde is associated with schizophrenia with severe psychomotor retardation (54). The authors of these studies indicate that IgM and IgA to TRYCATs are regulatory, so a reduced IgM response to TRYCATs may indicate downregulation of the TRYCATs pathway in schizophrenia (50).

The pool of natural antibodies includes antibodies with catalytic properties (55). Such antibodies bind and catalytically modify the antigen. Catalytic antibodies are only a minor part of natural antibodies but significantly enhance the immune system’s functionality (55). Catalytic antibodies hydrolyzing DNA (56), RNA and miRNA (57, 58), histones (59), and myelin basic protein (MBP) (60, 61) have been described in schizophrenia. Notably, these antigens are DAMPs. Therefore, on the one hand, their hydrolysis and removal can mitigate the immune response to these antigens. On the other hand, an increase in the level of catalytic antibodies is accompanied by immune dysfunction. Besides, some catalytic antibodies may be pathogenic. Indeed, MBP-hydrolyzing antibodies of autistic spectrum disorder patients have been shown to reduce long-term potentiation in the rat hippocampus (62). Consequently, further research into the role of natural catalytic antibodies in schizophrenia is needed.

### Cellular Components of the Innate Immune System

Leukocytes play a central role in the functioning of innate immunity. Leukocytes include natural killer cells, dendritic cells, neutrophils, macrophages, eosinophils, basophils, and monocytes. Summarized data on changes in the cellular components of the innate immune system are presented in Table 3. These changes will be discussed in more detail below.

**TABLE 3:** Quantitative changes in innate immunity cells in schizophrenia.

Immune cells	Changes	Risk of bias	References
NK	One meta-analysis shows an increase in both relative and absolute levels of CD56+ cells in patients with schizophrenia. However, there are case-control studies that suggest that the number of NK cells in patients with schizophrenia does not differ from healthy donors or even significantly lower.	Moderate	(59–61)
DC	One cross-sectional case-control study describing dendritic cells in schizophrenia suggests that their relative abundance is reduced in schizophrenia compared to healthy controls.	Unclear	(63)
Neutrophils	Three meta-analyses describe a significant increase in neutrophil levels and neutrophil/lymphocyte ratio in schizophrenia.	Low	(65–67)
Eosinophils and basophils	One meta-analysis indicates that in patients with schizophrenia, the absolute and relative number of eosinophils in the blood does not differ from healthy donors. One case-control study from the same year indicates a significant increase in blood eosinophil levels in patients with schizophrenia. Eosinophils were the only blood cells that were significantly reduced in women with schizophrenia compared to men with schizophrenia. According to the same meta-analysis, there is no quantitative change in basophils in patients with schizophrenia compared to healthy controls.	Eosinophils – moderate; Basophils – moderate	(67, 73, 74)
Monocytes	Three meta-analyses describe a significant increase in monocyte levels and monocyte/lymphocyte ratio in schizophrenia.	Low	(65–67)
Macrophages	The increase of absolute number of macrophages in schizophrenia in CSF, perivascular spaces, and subependymal zone.	Moderate	(87–90)

*NK, natural killer; CD, cluster of differentiation; DC, dendritic cell; CSF, cerebrospinal fluid.*

#### Natural Killers

Natural killers are cells of the innate immune system with solid cytolytic activity. These cells protect host against microbial agents and tumors (63). In the context of mental diseases and schizophrenia NK cells are rarely studied. One 2013 meta-analysis on lymphocytes in schizophrenia describes NK cells. The authors of the study demonstrate both a relative and absolute increase in the number of NK cells in patients with schizophrenia compared with healthy donors (64). This evidence has a moderate risk of bias (Table 2). However, later studies show contradictory results. For example, works published later than the meta-analysis (2018 and 2021, respectively) indicate that the level of NK cells may be lower than that of healthy donors (65) or not change at all (66). Tarantino et al. (66) also describe an increase in HLA-DR expression on activated CD56+ cells in patients with schizophrenia. The expression of this receptor positively correlates with the expression of activated NK cell group 2 isoform C (NKG2C) receptor (overexpression of this receptor is usually characteristic of viral diseases, especially cytomegalovirus infection). In turn, the NK cell-activating receptor also known as Nkp46 receptor or CD335 (one of the main inducers of the cytotoxic properties of NK cells) significantly decreased in patients with schizophrenia. It correlated negatively with the expression of HLA-DR.

#### Dendritic Cells

Dendritic cells are highly specialized antigen-presenting cells responsible for initiating the primary immune response (67). Studies describing dendritic cells in schizophrenia are practically not found. Only one case-control study of 2009 (68) demonstrates a significant relative decrease in dendritic cells compared to healthy donors.

#### Neutrophils

Neutrophils are among the most studied cells of the immune system in schizophrenia. The neutrophil-to-lymphocyte ratio is a good marker of immune system dysregulation in various diseases and is used as a prognostic criterion (69). The second major area of work is investigating the production of reactive oxygen species by neutrophils in schizophrenia. Three new meta-analyses (2019, 2020, and 2020, respectively) demonstrate an increase in the level of neutrophils in the blood of patients with schizophrenia and an increase in the neutrophil-to-lymphocyte ratio (70–72). Several studies describe an increase in neutrophil production of reactive oxygen species in schizophrenic patients (73–75). The evidence presented has a low risk of bias (Table 3). All of the above studies point to a possible active role of neutrophils in the pathogenesis of schizophrenia. Pinkhas S. et al. (73) states that bactericidal activity in patients with schizophrenia has not changed. Sometimes not quite usual correlations between neutrophils and manifestations of the disease are investigated. Fang S-H et Al. found that the higher the neutrophil-to-lymphocyte ratio in patients, the lower their quality and quantity of sleep (76).

#### Eosinophils and Basophils

Eosinophil activity is mainly associated with allergic reactions, fungal infections, and parasitic infestations (77). In this regard, studies of eosinophils in schizophrenia are pretty rare. A 2020 meta-analysis states that in all the studies reviewed, the level of eosinophils in the blood of patients with schizophrenia did not differ from the level in healthy donors (72). One case-control study, published the same year, describes a significant increase in the concentration of eosinophils in the blood of unmedicated schizophrenia patients (78). Jakobsen M. I. et Al. in 2018 published a comparison of the concentration of various immune cells in the blood of male and female patients with schizophrenia (79). The only statistically significantly different cells in the two groups were eosinophils. Women had significantly fewer of them than men. In 2008, a study was published stating that in patients with schizophrenia, the concentration of CCL11/Eotaxin, one of the proteins of eosinophil chemotaxis, significantly increases in the blood (80). In 2013, Fernandez-Egea E. et Al. found an increase in the same protein in the blood of young, healthy volunteers who use cannabis. Since there is a pattern between cannabis use and the incidence of schizophrenia, CCL11/Eotaxin may somehow be involved in the pathogenesis of schizophrenia (81). As for eosinophils, the main activity of basophils is associated with allergic reactions and multicellular parasites (82). Studies of basophils in schizophrenia are practically non-existent. One 2020 meta-analysis states that basophil counts are not significantly different from healthy donors (72). Overall, the evidence presented for no difference in eosinophils and basophils in schizophrenia has a moderate risk of bias (Table 3).

#### Monocytes

Monocytes are one of the key players in the inflammation process. It was previously believed that monocytes are a temporary link before their transformation into tissue phagocytes (83). Even though the transformation of monocytes into macrophage and dendritic subpopulations is still one of the essential functions of monocytes, it has now been established that these cells also have their immune activities in tissues. So, they can play the role of short-lived tissue effector cells (84). Three new meta-analyses (2019, 2020, and 2020, respectively) demonstrate an increase in the level of monocytes in the blood of patients with schizophrenia and an increase in the neutrophil-to-lymphocyte ratio (70–72). This evidence has a low risk of bias (Table 3). In addition to leaving the tissues and forming an increased pool of tissue phagocytes, monocytes in the blood of patients with schizophrenia also have their damaging activities. Kowalski J. et al. in 2001 described the increased production of pro-inflammatory cytokines such as IL-1β and TNF-α by monocytes (85). Beumer W. et al. (86) also show the increased content of circulating monocyte-associated pro-inflammatory cytokines in patients with schizophrenia.

#### Macrophages

An increased concentration of macrophages in patients with schizophrenia is found in the cerebrospinal fluid (87). In perivascular spaces (88, 89), and subependymal zone (90), CD163+ cells are found in significantly higher amounts in patients with schizophrenia than in healthy donors. Studies describe excessive production of macrophage-associated pro-inflammatory cytokines (86). Expression of the CD68 receptor on macrophages of schizophrenic patients does not change compared to healthy controls (91, 92).

## Impairments in Adaptive Immunity in Schizophrenia

### Changes in the Cellular Components of Adaptive Immunity

The main cellular components of adaptive immunity are T and B cells. Changes in their level are summarized in Table 4 and discussed in more detail below.

**TABLE 4:** Quantitative changes in adaptive immunity cells in schizophrenia.

Immune cells	Changes	Risk of bias	References
Lymphocyte (total)	One meta-analysis demonstrates the unchanged level of lymphocytes in patients with schizophrenia compared with healthy donors.	Moderate	(67, 94, 95)
B cells	Most of the studies analyzed in a systematic review indicate that the level of B cells in the blood of patients does not differ from healthy donors. Higher densities of CD20+ cells were observed in the hippocampus in residual and paranoid schizophrenia patients versus controls.	Moderate	(97, 98)
T cells	One meta-analysis states that in patients with schizophrenia, there is an increase in the absolute level of CD3+ and CD4+ cells, as well as an increase in the CD4/CD8 ratio. Increased T cell levels in CSF and hippocampus was observed.	Moderate	(59, 98, 100)

*CD, cluster of differentiation; CSF, cerebrospinal fluid.*

#### Lymphocytes (Total)

A 2020 meta-analysis (72) describes unchanged total lymphocyte counts in patients with schizophrenia compared with healthy donors. This evidence has a moderate risk of bias (Table 4). Studies not included in the meta-analysis demonstrate elevated levels of lymphocytes (93), and vice versa, more common lymphopenia in patients with schizophrenia (94).

#### B Cells

B cells are attracting the attention of researchers due to their ability to produce autoantibodies that may play a negative role in the pathogenesis of schizophrenia. Ezeoke A. et Al. in their systematic review found 20 different autoantibodies whose titers were significantly higher in patients with schizophrenia than in healthy donors (95). A 2019 systematic review (96) analyzed studies on B cells’ level in the blood of patients with schizophrenia. Sixteen of the twenty-two studies found no change in the blood concentration of B cells in patients with schizophrenia (two studies included only the group of patients on therapy), two studies found a decrease in B cells (in both studies, patients were medicated), and four – increase in concentration (two studies included only a group of patients were medicated). Increased CD20+ cell density has been found in the hippocampus of patients with schizophrenia (97). B cells may contribute to the pathogenesis of schizophrenia not so much by changing their number but by changing the composition of synthesized autoantibodies. For more information on autoantibodies in schizophrenia, see section “Changes in the Molecular Components of Adaptive Immunity: Antibodies and Autoimmunity in Schizophrenia.”

#### T Cells

The idea of T cell pathogenesis of schizophrenia first appeared in 1980 (6). These ideas turned into theory in 1992–1995 (7, 8). The ability of T cells to induce neuroinflammation and neurodegeneration maintains an interest in these cells in the context of the pathogenesis of schizophrenia (98). A 2013 meta-analysis (64) describes an increase in the absolute level of CD3+ and CD4+ cells and an increase in the CD4/CD8 ratio in the blood of patients with schizophrenia relative to healthy donors. This evidence has a moderate risk of bias (Table 4). An increase in T cell concentration has also been shown for CSF (99) and the hippocampus (97) of schizophrenic patients. Muller et al. in 1998 showed a significant increase in CD8+γ/δ+ lymphocytes (100). Mazzrello et al. in 2004 demonstrated a significant decrease in the percentage of CD8+ cells (101). Two 2007 case-control studies showed a decrease in T cell proliferative response (102, 103). In 2014 Ding et al. (104) demonstrated higher proportions of Th17 cells relative to healthy donors.

### Changes in the Molecular Components of Adaptive Immunity: Antibodies and Autoimmunity in Schizophrenia

Antibodies are the main effectors of the second line of defense of the immune system. Alterations in the level or reactivity of antibodies are associated with various pathologies. The available evidence for altered antibody levels or reactivity to microbial or self-antigens in schizophrenia is summarized in Table 5.

**TABLE 5:** Abnormalities in the level or reactivity of antibodies in schizophrenia.

Changes	Risk of bias	References
**Antibodies to microbial antigens**		
Patients with schizophrenia have elevated IgG and IgM levels against *Toxoplasma gondii.*	Low	(109–111)
The presence of antibodies to other pathogens including *Chlamydophila pneumoniae*, *Chlamydophila psittaci*, *Human Endogenous Retrovirus W*, *Human Herpesvirus 2*, and *Borna Disease Virus* is associated with a risk of schizophrenia.	Moderate	(112)
**Autoantibodies**		
An increased prevalence of anti-NMDAR antibodies has been found in schizophrenia.	Low	(121–123)
An increased prevalence of anti-GAD65 and anti-VGKC antibodies has been observed in schizophrenia.	Moderate	(129)
Elevated levels of anti-gluten, anti-gliadin, anti-transglutaminase 2, and anti-wheat antibodies were found in patients.	Moderate	(130)

*Ig, immunoglobulin; NMDAR, N-methyl-D-aspartate receptor; GAD, glutamic acid decarboxylase; VGKS, voltage-gated potassium channel.*

#### Antibodies to Microbial Antigens

Parasitic, bacterial, and viral infections are associated with the risk of developing schizophrenia. In a population-based register study of Benros M. E. et al., it was shown that hospitalization due to any infection increased the risk of schizophrenia by 60%, and in the presence of an autoimmune and infectious disease, the risk increased by 125% (105). Prenatal or childhood infections also increase the risk of schizophrenia (106, 107).

The most marked association of schizophrenia is shown with parasitic infection caused by *Toxoplasma gondii*. The meta-analysis by Torrey E. F. et al. showed that patients with schizophrenia had an increased prevalence of antibodies (primarily IgG class) against *Toxoplasma gondii* (108). According to another meta-analysis, 7.6% of individuals with acute psychosis had IgM antibodies to *Toxoplasma gondii*, and the risk of detecting such antibodies was 1.7-fold higher compared to healthy controls (109). The association was stronger in chronic schizophrenia than in first-episode psychosis. An association with a geographic region is also revealed. In a meta-analysis by Sutterland A. L. et al., an association with *Toxoplasma gondii* infection has been confirmed for schizophrenia as well as bipolar disorder, obsessive-compulsive disorder, and addiction, but not for major depression (110). Thus, evidence for an association of schizophrenia with *Toxoplasma gondii* infection has a low risk of bias (Table 5). A statistically significant association of schizophrenia (including the presence of antibodies to relevant pathogens) has been found with infections other than *Toxoplasma gondii*, including *Chlamydophila pneumoniae*, *Chlamydophila psittaci*, Human Endogenous Retrovirus W, Human Herpesvirus 2, and Borna Disease Virus (111). Deficit schizophrenia was associated with seropositivity for antibodies to cytomegalovirus (112). But this evidence was moderately biased (Table 5). Nevertheless, association does not imply causation, so studies on the role of antibodies to microbial antigens in the etiopathogenesis of schizophrenia are needed.

#### Autoantibodies

There is no convincing evidence that schizophrenia is an autoimmune disease. However, some of the observations found in psychosis are consistent with modified Witebsky’s postulates of autoimmune pathogenicity, suggesting that illness in some subgroups of patients has an autoimmune component (113). Some studies indicate that schizophrenia is associated with an increased prevalence of autoimmune diseases and the presence of autoantibodies. Schizophrenia has been associated with several autoimmune diseases, including autoimmune hepatitis, multiple sclerosis, rheumatoid arthritis, systemic lupus erythematosus, autoimmune thyroiditis, Graves’ disease, type 1 diabetes, Guillain-Barré syndrome, and psoriasis (114). These diseases significantly increase the risk of developing schizophrenia and vice versa. Conversely, the risk of subsequent autoimmune diseases is significantly increased in schizophrenia, especially with concomitant infectious pathology (115).

Infections, antigenic mimicry, and other unknown causes contribute to the breakdown of Infections, antigenic mimicry, and other unknown causes contribute to auto-tolerance breakdown, the activation of autoimmune mechanisms, and the generation of autoantibodies in schizophrenia. Environmental toxic effects, metabolic disorders, oxidative stress can also cause changes in the antigenic properties of self-molecules in schizophrenia (116). This can lead to the recognition of autoantigens by the immune system and the generation of autoantibodies. Ezeoke A. et al. showed in a systematic review that an increase in the prevalence of 20 different autoantibodies was observed in patients with schizophrenia compared with controls (95). In particular, antinuclear antibodies, anti-cardiolipin IgG and IgM, anti-double-stranded DNA (dsDNA), and anti-single-stranded DNA (ssDNA), anti-gliadin, anti-histone, and other autoantibodies have been found. Nevertheless, marked study heterogeneity and inconsistency have been observed.

Patients with various forms of psychosis showed the presence of antibodies to multiple neuroantigens, including ionotropic glutamate receptors (N-Methyl-D-Aspartate Receptor (NMDAR) and α-amino-3-hydroxy-5-methyl-4-isoxazolepropionic acid receptor (AMPAR)), dopamine D2 receptors, metabotropic GABA receptors, voltage-gated potassium channels, M1 and M2 cholinergic receptors (117–119). One early meta-analysis showed that 7.98% of patients with schizophrenia were anti-NMDAR antibody positive. Of these, 1.46% had antibodies of the IgG subclass (120). Another meta-analysis showed that elevated titers of anti-NMDAR antibodies in patients with schizophrenia were detected three times more often than in healthy people (121). Antibodies of various classes (IgG, IgM, and IgA) against NR1, NR1/NR2B, and NR2A/NR2B subunits were found in patients. Also, NR2A/NR2B antibody levels were significantly higher in patients with first-episode psychosis than in healthy controls (121). Evidence-based umbrella review also confirms increased NMDAR seropositivity in schizophrenia (122). Thus, the evidence for increased anti-NMDAR seropositivity in schizophrenia has a low risk of bias (Table 5).

In general, results on anti-NMDAR antibodies in schizophrenia varied significantly. Some studies failed to detect anti-NMDAR antibodies in any patient with psychosis (123), while others reported high seropositivity in both patients with psychosis (9,4%) and healthy controls (8,5%) (124). More recently, Cullen A.E. et al. reported that this heterogeneity in results was significantly associated with assay type, illness stage and study quality (125). The use of a live cell-based assay resulted in higher estimates of anti-NMDAR antibody prevalence. The prevalence of anti-NMDAR seropositivity was more elevated in exclusively first-episode psychosis samples than in mixed or multi-episode samples. Therefore, it is necessary to standardize assay procedures and consider clinical factors to obtain more accurate estimates of anti-NMDAR antibody prevalence in schizophrenia.

Anti-NMDAR antibodies are biomarkers for autoimmune encephalitis. Detection of anti-NMDAR antibodies in subgroups of patients without neurological symptoms made it possible to identify a new nosological entity, autoimmune psychosis (126). There is evidence that anti-NMDAR antibodies can induce internalization and reduce the density of surface NMDARs expressed in hippocampal neurons (127). Further research is needed to identify the role of anti-NMDAR antibodies in provoking psychotic symptoms.

Other anti-neuronal antibodies have been found in schizophrenia. Meta-analysis showed that a prevalence of autoantibodies to glutamic acid decarboxylase (GAD65) was 4.6% in schizophrenia compared to 2.7% in healthy controls (128). The prevalence of antibodies to the voltage-gated potassium channel (VGKC) complex was 1.5% in psychoses compared to 0.7% in healthy controls (128). This evidence has a moderate risk of bias (Table 5). Other circulating IgG autoantibodies have also been detected in schizophrenia (129). It is also shown that the treatment affects the level of antibodies. The generation of autoantibodies may be associated with an impaired blood-brain barrier in schizophrenia. This is indicated by the results of a recent meta-analysis (130). An increased level of total protein in the cerebrospinal fluid (CSF) and elevated CSF/serum albumin ratio was revealed in schizophrenia.

Some evidence suggests an association between gluten sensitivity and schizophrenia (Table 5). A meta-analysis showed that biomarkers of gluten sensitivity (anti-gluten, IgG and IgA to gliadin, anti-transglutaminase 2 IgA, and anti-wheat antibodies) were elevated in various forms of psychosis compared with healthy donors (131). This is consistent with the results of other work that examined the reactivity of antibodies toward gluten proteins in schizophrenia (132). It was shown that the reactivity of antibodies to gluten in patients with schizophrenia differed from that in people with celiac disease. In particular, gluten-sensitive schizophrenic patients did not show elevated levels of anti-transglutaminase 2 IgA. Thus, the immune response to gluten in schizophrenia and celiac disease is different.

Generally, antibodies with different reactivity have been found in schizophrenia, but their pathological functions remain elucidated.

## Changes in the Immune System Organs Associated With Schizophrenia

The previously described changes in molecular and cellular immunity may reflect the pathology of the immune system organs in schizophrenia. However, studies at the level of immune system organs are limited. Available data are summarized in Table 6.

**TABLE 6:** The immune system organs anomalies associated with schizophrenia.

The immune system organ	Changes	Risk of bias	References
Bone marrow	Several clinical cases have been described of the effect of stem cell or bone marrow transplantation on psychotic symptoms, as well as the association of megaloblastic anemia with psychosis.	Unclear	(133–135)
Thymus	There are no data directly for schizophrenia.	Unclear	–
Lymph nodes, lymphatic and glymphatic system	Changes in the functional activity of the lymphoid tissue of the inguinal lymph nodes were observed in patients with schizophrenia.	Unclear	(146)
Spleen	Decreased expression of the colony stimulating factor 1 receptor (CSF1R) was found in the spleen but not in the cerebellum and parietal cortex in schizophrenia compared with controls.	Unclear	(147)
Liver	The prevalence of chronic liver diseases in schizophrenia is significantly higher than in the general population, but the risk of liver cancer is lower.	Low	(149–152)
	Changes in the expression of several genes including BDNF and Apolipoprotein A1 were found. Antipsychotics also disrupt gene expression in the liver in schizophrenia.	Unclear	(153–155)
	Air pollution are associated with disruption of the gut microbiome and liver dysfunction in schizophrenia.	Unclear	(159)
Intestines and gut-associated lymphoid tissue	An increased serum zonulin levels have been observed in schizophrenia patients, which may contribute to increased intestinal permeability.	Unclear	(161, 162)
	Significant changes in the microbiota composition were found in patients with schizophrenia compared with non-psychotic controls.	Low	(166–169)

*BDNF, brain-derived neurotrophic factor.*

The bone marrow is the site of hematopoietic stem cells that generate all immune cells. The first mention of bone marrow anomalies in schizophrenia was published in 1964 (133). But since then, there have been few studies of bone marrow in schizophrenia, which are represented mainly by clinical cases. There is an intriguing report of the induction of severe chronic psychosis after allogeneic stem cell transplantation from a donor with schizophrenia (134). In another case, persistent remission of psychotic symptoms in treatment-resistant schizophrenia after bone marrow transplantation from a healthy donor is reported (135). There is also evidence that severe megaloblastic anemia may be accompanied by psychotic symptoms (136). These data may indicate the involvement of bone marrow anomalies in the pathogenesis of schizophrenia, but further research is required to identify specific mechanisms and pathways. However, some changes may be associated with antipsychotic therapy. Some antipsychotics (olanzapine, clozapine, phenothiazines, etc.) are known to cause agranulocytosis, neutropenia, monocytopenia, and thrombopenia (137–139).

The thymus is the second most crucial primary organ of the immune system in which T cell lymphopoiesis occurs. The T cell abnormalities outlined in Section “T Cells” may be associated with thymus dysfunction. Kinney et al. proposed the hypothesis of abnormal immune system development and thymus dysfunction in schizophrenia (140, 141). This hypothesis suggests that schizophrenia is associated with pre- or perinatal exposure to adverse factors that cause latent immune vulnerability. The manifestation of this vulnerability is related to changes in immune function and thymus involution after puberty. Individuals become more susceptible to infections that contribute to schizophrenia (140, 141). However, there are no studies of the thymus in schizophrenia, and this hypothesis requires experimental confirmation. Patients with 22q11.2 deletion syndrome can help reveal the immunological basis of thymus dysfunction and psychosis. With a variety of clinical manifestations, including congenital heart disease, velopharyngeal insufficiency, and recurrent infections, such patients have thymic hypoplasia and suffer from psychotic symptoms (142, 143). Moreover, about 30% of patients with this syndrome develop schizophrenia, with significantly increased risk (about 12 to 80 folds) than the general population (144). There is evidence of an increased percentage of Th17 in adults with psychotic symptoms compared with non-psychotic adults with 22q11.2 deletion syndrome (145). These data highlight the importance of Th17 cells in developing psychiatric symptoms.

The glymphatic system and meningeal lymphatic vessels are involved in removing waste products from the central nervous system. The meningeal lymphatic vessels drain waste products from the cerebrospinal and interstitial fluid to the deep cervical lymph nodes. Impairment of meningeal lymphatic function induced cognitive impairment in mice (146). The cognitive and social deficits observed in schizophrenia may also be associated with lymphatic dysfunction. There is evidence of a change in the functional activity of the lymphoid tissue of the inguinal lymph nodes in patients with schizophrenia (147). In particular, a higher amount of blast forms of the lymphocytes and activated lymphocytes and a high pynocytose activity of the reticular cells and macrophages was found in schizophrenia. But this study turned out to be the only one directly related to schizophrenia, so further research is needed.

Like the lymph nodes, the spleen is a secondary organ of the immune system. Studies of the spleen and its function in schizophrenia are also rare. Decreased expression of the colony-stimulating factor 1 receptor (CSF1R) was found in the spleen but not in the cerebellum and parietal cortex in schizophrenia compared with controls (148). CSF1R interacts with the transcription factor PU.1 (SPI1) and regulates microglia and macrophages’ proliferation, differentiation, and survival. The SPI1 levels in the cerebellum and spleen were significantly higher in the schizophrenia group than in the control group (148). There is also evidence that clozapine affects the expression of activity-dependent neuroprotective protein 2 (ADNP2) in the spleen of mice, which may indicate treatment-induced autophagy dysfunction. These data may indicate the existence of the brain-spleen axis, whose dysregulation may contribute to schizophrenia.

The liver is also involved in immunity as it synthesizes acute phase proteins and contains various subpopulations of lymphocytes. There is a hypothesis about liver dysfunction and schizophrenia (149). Patients with schizophrenia exhibited a significant prevalence of chronic liver diseases than those in the general population (150, 151). The meta-analysis also found an approximately 3-fold increase in the risk of hepatitis B and C in patients with schizophrenia (152). The prevalence of hepatitis B and C in schizophrenia was 6 and 7%, respectively. But at the same time, according to a meta-analysis, patients with schizophrenia have a lower risk of liver cancer (153). Liver dysfunction in schizophrenia may be associated with changes in gene expression. For example, BDNF expression levels from the liver in schizophrenic patients were significantly higher than in healthy controls (154). Interestingly, there were no differences in BDNF expression in the spleen. A negative correlation was observed between mature BDNF in the parietal cortex and mature BDNF in the liver. Apolipoprotein A1 levels were reduced in the liver as well as in the brain, red blood cells, cerebrospinal fluid, and serum of schizophrenic patients (155). In another study, 14 proteins were shown to be significantly altered in liver samples from schizophrenia patients compared to healthy controls, with some of these proteins being related to oxidative stress (156). Typical and atypical antipsychotics are also known to alter gene expression in the liver of patients (157). Therefore, dysregulation of the brain-liver axis may be associated with schizophrenia.

Liver dysfunction could potentially be related to the gut microbiome (158). According to the “gut-liver” axis concept, a shift in gut microbiota composition can result in dysfunction of the gut mucosal barrier (“leaky gut” syndrome). This, in turn, can lead to increased translocation of bacteria to the liver *via* the portal vein, activation of the immune response, and migration of immune cells to the liver, which likely plays a crucial role in liver dysfunction (159). Interestingly, air pollution and long-term NO2 exposure are associated with disruption of the gut microbiome and liver dysfunction in schizophrenia (160).

Increased intestinal permeability (“leaky gut” syndrome) leading to the inappropriate access of antigens to the gut-associated lymphoid tissue plays an essential role in peripheral and neuroinflammation (161). Increased leakage of the tight epithelial junctions of the intestine in schizophrenia may be associated with changes in zonulin (pre-haptoglobulin 2) concentrations. Zonulin is a protein that increases small intestine permeability and contributes to innate intestinal immunity. Remarkably, increased serum zonulin levels have been observed in schizophrenia patients (162, 163). Also, significant associations of gene polymorphisms of haptoglobulin (a product of zonulin processing) are found in schizophrenia (164).

As an essential constituent of the mucosal immune system, gut-associated lymphoid tissue may also be associated with the development of schizophrenia. Usually, immune cells in gut-associated lymphoid tissue can be activated in response to both specific antigens and microbial signals, including lipopolysaccharide, flagellin, or DNA. The microbiota also influences immune cells through metabolic products such as tryptophan metabolites and short-chain fatty acids. For example, short-chain fatty acids activate and maintain Treg cells through G-protein-coupled receptors (165). Several studies indicate that activation of brain autoreactive T cells occurs in the gut-associated lymphoid tissue (166). On the one hand, resting circulating autoreactive T cells receive positive stimulatory signals from particular microbial subsets of the gut microbiota. On the other hand, a deficiency in inhibitory signals from luminal bacteria impairs local Treg induction and function, promoting autoreactive T cells’ activation (166). Significant changes in the microbiota composition (including mycobiota and phageome) were found in the oropharynx and intestines of patients with schizophrenia compared to non-psychiatric controls (167–171). Moreover, microbiota dysregulation or dysbiosis has been associated with behavioral anomalies in rodent studies (172). Notably, antipsychotic treatment may contribute to gut microbiome alteration (173). Evidence for microbiota dysregulation in schizophrenia has a low risk of bias (Table 6). Thus, gut microbiota dysregulation in schizophrenia may impair the gastrointestinal barrier, immune homeostasis, brain development, and neurotransmission, which is associated with chronic low-grade inflammation and the formation of autoreactive T cells (161, 166, 174). Therefore, the microbiota-gut-brain axis dysregulation should be considered as an essential factor in the pathogenesis of schizophrenia [for more information, see (175–177)].

Altogether, some changes in the organs of the immune system are characteristic of schizophrenia. But studies of the immune system at the organ level are rare and more research is needed.

## Immune-Associated Alterations in the Brain

Activation of peripheral immunity affects the central nervous system and contributes to neuroinflammation in schizophrenia (178). Conversely, neuroinflammation, such as that caused by neuroinfection, induces a systemic pro-inflammatory state (179). Overall, the role of peripheral inflammation in the pathophysiology of schizophrenia is well supported by multiple findings of elevated inflammatory markers in the brains of patients (Table 7). The results of postmortem studies indicate an increase in the expression of many pro-inflammatory genes, including Serpin family A member 3 (SERPINA3) and Interferon-induced transmembrane protein (IFITM) in the brains of schizophrenic patients (180, 181). A recent meta-analysis also showed an increase in pro-inflammatory gene expression at the transcript (TNFα, IL1β, IL6, and IL8) and protein (TNFα and IL1β) levels in the brains of schizophrenic patients compared to healthy individuals (182). These changes may be associated with increased activity of nuclear factor kappa B (NF-κB), which regulates the expression of pro-inflammatory factors (183, 184). Higher NF-κB activity associated with increased expression of NF-κB signaling pathway components found in the prefrontal cortex of patients with schizophrenia (183). These data has a low risk of bias (Table 7). Besides, changes in density and signs of microglia activation were found in the brains of patients. According to a 2015 systematic review, four studies state that microglial cell density increases, while three studies indicate cell density does not change (185). PET studies of microglial activation in patients with schizophrenia have found increased cell activation in the early stages of the disease at the most severe symptomatology (185). A 2019 meta-analysis describes an increase in 18-kDa translocator protein (TPSO) in patients with schizophrenia compared to healthy controls (186). Although there is evidence that central low-grade inflammation in schizophrenia is not always accompanied by increased TSPO expression or ligand binding (187). Also, several studies describe an increase in HLA-DR expression on microglial cells in patients with schizophrenia (91, 188). In some studies, no differences in HLA-DR expression are found (189, 190). Thus, the evidence for microglial activation in schizophrenia has moderate risk of bias (Table 7). Another line of evidence indicates immune cells infiltration of brain tissue (88, 191, 192). Perivascular macrophages (CD163+) have been identified in the brain parenchyma of patients in about 40% of cases of schizophrenia with “severe inflammation” (88). This is associated with increased levels of soluble intercellular adhesion molecule-1 (sICAM1) in serum, mRNA expression of ICAM1 and VE-cadherin in the brain, and enrichment of ICAM1 in the brain endothelium (88). Besides, an increased density of CD3+ T-lymphocytes and CD20+ B-lymphocytes was found in some brain areas in about 1/3 of patients with schizophrenia (191, 192). All these data indicate neurovascular endothelial dysfunction and hyperpermeability of the blood-brain barrier in schizophrenia (193), which can be caused, among other things, by peripheral immune abnormalities.

**TABLE 7:** Immune-associated changes in the brain of schizophrenia patients.

Changes	Risk of bias	References
The expression of pro-inflammatory genes at the transcript and protein level in the brain of some subgroups of patients is increased.	Low	(181, 182)
The changes in density and signs of microglia activation were found in the brains of patients.	Moderate	(185, 186)
Increased infiltration of perivascular macrophages, T- and B-lymphocytes has been identified in some areas of the brain in certain subgroups of patients.	Unclear	(88, 191, 192)
Approximately 1/3 of patients have signs of severe inflammation and can be classified into a separate group based on central and peripheral markers.	Unclear	(197, 198)
Peripheral and neuroinflammation is associated with cognitive and neuroanatomical alterations	Unclear	(197–203)

It should be especially emphasized that signs of neuroinflammation are observed only in certain subgroups of patients. For example, an increase in the expression of pro-inflammatory genes is observed in only about 40% of patients (180). Infiltration of brain tissue by immune cells was also observed in 1/3 of patients (191, 192). Signs of peripheral inflammation and elevated cytokine levels are found in only a subset of schizophrenia patients who are linked to poorer response to antipsychotic treatment (178, 194). Combining data on the levels of peripheral inflammatory markers and post-mortem brain markers using cluster analysis methods, it was found that approximately 30 to 50% of patients with schizophrenia can be classified to a subtype with increased levels of inflammation (195–197). Interestingly, inflammatory subtypes are observed in both antipsychotic naïve first-episode schizophrenia patients (198) and chronic psychosis spectrum patients (197). Moreover, the proportion of patients with a severe inflammatory phenotype is approximately the same for first-episode and chronic patients and is about 36% (197, 198). Thus, subgroups with high inflammation are distinguished among patients.

Importantly, peripheral and neuroinflammation is associated with cognitive and neuroanatomical alterations. Patients with chronic psychosis in the high inflammatory subtype had greater cortical thickness and subcortical volumes than individuals in the low inflammatory subtype (197). Individuals with first-episode schizophrenia in the high inflammatory subtype had greater thickness in the right parahippocampus, bank superior sulcus, and caudal anterior cingulate compared to patients in the low inflammatory subtype (198). Neuroinflammation is also associated with brain white matter pathology, as the density of orbitofrontal white matter neurons was increased in patients with high levels of inflammation (199). Also, plasma CRP levels were inversely correlated with the thickness of the prefrontal cortex of patients (200). Moreover, patients with elevated peripheral inflammatory markers suffer from more severe cognitive impairments than patients without active inflammation (201, 202). For example, patients with elevated serum CRP showed significantly worse working memory than controls (200). Additionally, chronic patients in the high inflammatory subtype had impairments in inhibitory behavioral control and visuospatial working memory (197). Peripheral inflammation measured by highly sensitive CRP levels is also associated with changes in brain perfusion and with psychotic symptom scores (203).

Together, these data indicate that peripheral immune alterations influence the central nervous system and contribute to the pathogenesis of schizophrenia. These findings may also be associated with endothelial dysfunction and hyperpermeability of the blood-brain barrier (193).

## Overview of the Immune System Abnormalities in Schizophrenia

**FIGURE 3:**
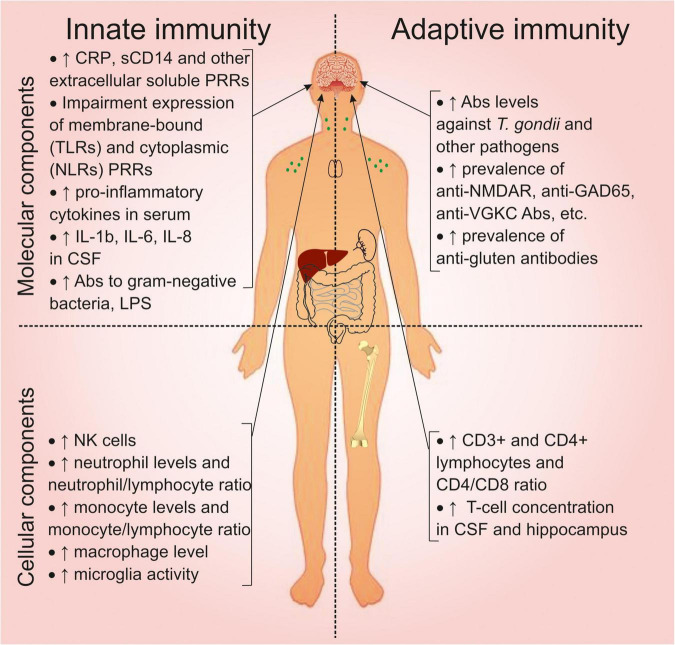
Overview of the immune system abnormalities associated with schizophrenia. CRP, C-reactive protein; CD, cluster of differentiation; PRRs, pattern recognition receptors; TLRs, toll-like receptors; NLRs, (NOD)-like receptors; IL, interleukin; CSF, cerebrospinal fluid; LPS, lipopolysaccharide; NK, natural killer; NMDAR, N-methyl-D-aspartate receptor; GAD, glutamic acid decarboxylase; VGKC, voltage-gated potassium channel.

We have summarized the previously described abnormalities of the immune system in schizophrenia in Figure 3. Changes in the molecular components of innate immunity are associated with activation of the inflammatory response (Section “Molecular Components of the Innate Immune System”). This is associated with increased extracellular soluble PRRs and abnormal expression of membrane-bound and cytoplasmic PRRs. Activation of the innate immune response is reflected in an increase in pro-inflammatory cytokines in serum and cerebrospinal fluid. Changes in the molecular components of adaptive immunity (Section “Changes in the Molecular Components of Adaptive Immunity: Antibodies and Autoimmunity in Schizophrenia”) are associated with increased levels of antibodies to *T. gondii* and other pathogens and an increased prevalence of anti-NMDAR and anti-gluten antibodies. Dysbiosis and increased intestinal permeability are associated with natural antibodies to gram-negative bacteria and LPS.

Abnormalities in the cellular component of innate immunity (Sections “Cellular Components of the Innate Immune System” and “Changes in the Cellular Components of Adaptive Immunity”) are associated with increased levels of NK cells, neutrophils, monocytes, macrophages, and microglia activation. The cellular components of adaptive immunity are characterized by increased CD3+ and CD4+ lymphocytes and T cells in the cerebrospinal fluid. Although the quantitative changes in cells are within the reference values.

All these changes indicate activation of the peripheral immune system. This in turn promotes neuroinflammation, which leads to cognitive and neuroanatomical alterations (Section “Immune-Associated Alterations in the Brain”). However, only a certain subtype (30 to 50% of all patients) exhibits inflammatory phenotype.

Thus, schizophrenia is accompanied by abnormalities in all immune system components.

## Usefulness of Immunological Data in Psychiatric Clinical Practice: Stratification of Patients

Schizophrenia is a heterogeneous disease in clinical manifestations and laboratory tests, including immune parameters. The current clinical diagnosis of schizophrenia does not consider the disease heterogeneity and does not allow individualized treatment prescriptions based on neurobiology. As stated above (Section “Immune-Associated Alterations in the Brain”), only some subsets of patients show signs of peripheral and neuroinflammation and respond poorly to antipsychotic treatment (194, 204). Therefore, immunological data may help stratify patients and identify subgroups with distinct phenotypes. However, the usefulness of a single immune-inflammatory marker for patient stratification may be low (205), so multiple parameters should be used. Several attempts have been made to stratify patients with psychosis and identify different endophenotypes based on clinical features (206). However, other researchers suggest that all clinical and biological data should be considered.

There are studies using inflammation-profiling approaches to stratify patients by level of inflammation (204, 207–210). For example, a recent study showed that using data on mRNA transcript levels of 56 immune genes using machine learning algorithms can distinguish between people with first-episode psychosis, chronic psychosis, and controls with >80% accuracy (210). The OPTiMiSE cohort study showed that uncontrolled clustering allowed the identification of clinical subtypes of first-episode psychosis patients (207). For one of the subtypes, lower levels of IL-15, higher levels of the chemokine ligand CXC (CXCL) 12, and prior exposure to cytomegalovirus were shown to be associated with a lack of remission.

Maes M. and colleagues proposed a hypothesis about the Immune-inflammatory response system (IRS) and the Compensatory Immune-Regulatory Reflex System (CIRS) in schizophrenia (50). An increase in the levels of various acute-phase proteins and cytokines/chemokines is associated with the activation of the IRS in schizophrenia. In contrast, CIRS aims to reduce inflammatory and immune responses and includes IL-1 receptor antagonist (sIL-1RA), sIL-2R and tumor necrosis factor-α receptors, Th-2 and Treg producing IL-4 and IL-10 and other components with immunoregulatory and anti-inflammatory effects. Clinical endophenotypes of schizophrenia differ in IRS and CIRS parameters. Therefore, immunological data on changes in IRS and CIRS parameters may better differentiate specific endophenotypes of schizophrenia. Moreover, Maes M. and colleagues propose to combine clinical and molecular immunological data, i.e., use all sets of traits to divide patients into subgroups with different phenotypes. They are supposed to use the nomothetic network approach (211).

Thus, immune-inflammatory data may help identify specific patient subgroups and disease biotypes (212). The use of clustering algorithms and machine learning methods will improve the accuracy of patient stratification (213). This stratification of patients with schizophrenia using inflammatory biosignatures and machine learning may also help identify subgroups of individuals who well respond to anti-inflammatory therapy, especially among antipsychotic-resistant patients.

## Unresolved Issues and Promising Future Research Directions for the Immune System in Schizophrenia

This systematic review identified gaps in knowledge about immune abnormalities in schizophrenia. In schizophrenia, soluble PRRs (CRP, sCD14, etc.) have been well studied. However, studies on membrane-bound and cytoplasmic PRRs are rare and controversial. Therefore, it is necessary to focus on changes in the expression level and functional activity of PRRs in schizophrenia. Unraveling these mechanisms will help develop new therapeutic strategies to influence inflammatory responses in schizophrenia. The study of the function of complement system proteins in the brain may also have translation perspectives. The functional role of the detected autoantibodies in schizophrenia is not fully understood. Further research is needed to elucidate the contribution of anti-NMDAR antibodies to the pathogenesis of schizophrenia. Complex changes in cytokine networks may reflect the heterogeneity of schizophrenia. The use of machine learning methods will help unravel this complex interaction in schizophrenia. The cellular components of the immune system are also impaired in schizophrenia, but the reasons for this remain elusive. The bone marrow is the source of all immune cells, so the study of hematopoietic stem cells can shed light on the causes of altered immune cell function. Studies of other immune organs can also help elucidate the state of the immune system in schizophrenia.

## Limitations

There are some limitations in the present systematic review. Firstly, it cannot be unequivocally stated that all published works on the topic of the review have been studied by the authors. Some relevant works could escape the attention of the authors. Secondly, meta-analyses and systematic reviews were frequently used in this systematic review, which may lead to the accumulation of Type I errors (214). Thirdly, the bias rating scale was developed by the authors of this work based on the scale from the study by Tanaka et al. (16); this scale may itself be biased.

## Conclusion

Schizophrenia is associated with abnormalities in all immune system components: from innate to adaptive immunity and from humoral to cellular immunity. Changes in the immune system indicate the activation of inflammatory responses in about 1/3 of patients with schizophrenia. Peripheral immune abnormalities contribute to neuroinflammation, which is associated with cognitive and neuroanatomical alterations and contributes to the pathogenesis of schizophrenia. Immunological parameters may help identify subgroups of individuals who well respond to anti-inflammatory therapy.

## Data Availability Statement

The original contributions presented in the study are included in the article/supplementary material, further inquiries can be directed to the corresponding author/s.

## Author Contributions

EE conceptualized the review. EE and MM searched the literature, critically analyzed, and wrote the draft manuscript. VB and SI interpreted and discussed the results. All authors contributed to the article and approved the final manuscript.

## Conflict of Interest

The authors declare that the research was conducted in the absence of any commercial or financial relationships that could be construed as a potential conflict of interest.

## Publisher’s Note

All claims expressed in this article are solely those of the authors and do not necessarily represent those of their affiliated organizations, or those of the publisher, the editors and the reviewers. Any product that may be evaluated in this article, or claim that may be made by its manufacturer, is not guaranteed or endorsed by the publisher.
